# Mean Corpuscular Volume as a Prognostic Marker in Patients with Non-Small Cell Lung Cancer Undergoing Surgical Resection: A Cohort Study

**DOI:** 10.3390/medicina62020395

**Published:** 2026-02-18

**Authors:** Soomin An, Wankyu Eo

**Affiliations:** 1 Department of Nursing, Dongyang University, Yeongju-si 36040, Republic of Korea; 2 Department of Internal Medicine, College of Medicine, Kyung Hee University, Seoul 05278, Republic of Korea

**Keywords:** carcinoma, non-small cell lung, mean corpuscular volume, pulmonary surgical procedures

## Abstract

*Background and Objectives*: Anatomical staging alone insufficiently explains survival heterogeneity in patients with resected non-small cell lung cancer (NSCLC). Although inflammation-based biomarkers have demonstrated prognostic value, the clinical relevance of erythrocyte-derived indices—particularly mean corpuscular volume (MCV)—remains poorly characterized in this setting. This study evaluated the prognostic significance of preoperative MCV and examined whether its integration with the Noble and Underwood (NUn) score improves survival prediction. *Methods*: We retrospectively analyzed patients with stage I–IIIA NSCLC who underwent complete surgical resection. Associations between clinicopathological variables and overall survival (OS) were assessed using Cox proportional hazards models. Prognostic performance was evaluated using the concordance index and the integrated time-dependent area under the curve. Continuous variables were modeled on their original scale without dichotomization. *Results*: Model comparison using the Akaike Information Criterion indicated that incorporation of the composite NUn–MCV index into the intermediate model—comprising age, basal metabolic rate, American Society of Anesthesiologists physical status, pleural invasion, and pathological stage—provided a superior model fit compared with inclusion of the NUn score and MCV as separate covariates. On this basis, the composite NUn–MCV model was selected as the full model. Across all evaluations, the full model demonstrated consistently greater discriminative ability for survival prediction than both the intermediate model and the baseline model based solely on pathological stage. *Conclusions*: Preoperative MCV independently predicts OS in patients with resected stage I–IIIA NSCLC. Integration of MCV with the NUn score into a composite index provides incremental prognostic value beyond anatomical staging and established clinical factors, supporting its use as a complementary tool for postoperative risk stratification.

## 1. Introduction

Lung cancer, comprising small cell lung cancer and non-small cell lung cancer (NSCLC), remains the leading cause of cancer-related mortality worldwide, accounting for approximately 1.82 million deaths—representing 18.7% of all cancer deaths—in 2022 [1].

NSCLC constitutes nearly 85% of all lung cancer diagnoses and encompasses several histologic subtypes, most commonly adenocarcinoma (approximately 40%), followed by squamous cell carcinoma (25–30%) and large cell carcinoma (10–15%) [2]. Despite advances in diagnostic and therapeutic strategies, nearly 70% of patients present with advanced or metastatic disease at the time of diagnosis [3].

Management of NSCLC is fundamentally stage dependent. Since its introduction by Pierre Denoix in 1953, the tumor–node–metastasis (TNM) classification has served as the central framework for disease staging and treatment decision-making in NSCLC [4]. For patients with stage I–II and select N2-negative stage IIIA NSCLC, curative treatment is most commonly achieved through surgical resection, with lobectomy representing the standard approach for operable candidates [1,2].

Despite the central role of pathological stage in guiding treatment and prognostic assessment in NSCLC [5], the wide dispersion of survival outcomes among stage-matched patients highlights the insufficiency of anatomy-based systems in representing tumor biology and host susceptibility [6]. Accordingly, sustained interest in identifying readily available, biologically informative biomarkers that complement conventional staging systems and enable more individualized prognostic stratification exists.

Clinical variables such as age, smoking history, and performance status provide incremental prognostic information but insufficiently reflect the systemic and biological heterogeneity underlying long-term outcomes in patients with NSCLC [7].

Increasing evidence supports the prognostic relevance of systemic inflammation in NSCLC. In this context, biochemical parameters (e.g., albumin and C-reactive protein [CRP]) and inflammation-based leukocyte ratios, such as the lymphocyte-to-monocyte ratio (LMR), neutrophil-to-lymphocyte ratio (NLR), and monocyte-to-lymphocyte ratio (MLR), have consistently been associated with long-term survival outcomes [8,9,10,11,12]. Composite indices integrating multiple inflammatory components—such as the Noble and Underwood (NUn) score, CRP-to-lymphocyte ratio (CLR), CRP–albumin–lymphocyte (CALLY) index, hemoglobin–albumin–lymphocyte–platelet (HALP) score, systemic inflammation response index (SIRI), and systemic immune-inflammation index (SII)—further enhance prognostic discrimination by capturing distinct yet complementary dimensions of the host inflammatory response [13,14,15,16,17,18]. Taken together, these observations highlight systemic inflammation as a fundamental determinant of oncologic outcomes.

In contrast, red blood cell (RBC)-derived indices have received comparatively limited attention in NSCLC prognostication. Malignancy is frequently accompanied by anemia, chronic inflammation, metabolic stress, and nutritional derangements, all of which can influence erythrocyte morphology and function [19,20,21]. Such alterations may reflect not only cancer-related anemia but also broader systemic physiological stress. Previous studies have shown that red-cell distribution width (RDW) and the hemoglobin-to-RDW ratio are associated with survival in NSCLC [22,23,24,25,26,27,28], suggesting that erythrocyte indices may serve as sensitive markers of host vulnerability and disease severity.

Mean corpuscular volume (MCV), a routinely reported parameter of complete blood count (CBC), has emerged as a potential prognostic biomarker owing to its universal availability, low cost, and ability to reflect erythroid stress, nutritional status, and systemic metabolic disturbances. Elevated MCV is observed across diverse clinical contexts, including vitamin B_12_ or folate deficiency, liver disease, hypothyroidism, alcohol exposure, smoking, advanced age, and myelodysplastic syndromes [29]. Increased MCV has been associated with higher all-cause and cancer-related mortality [30], as well as adverse outcomes in several malignancies, including head and neck, esophageal, gastroesophageal, and colorectal cancers [31,32,33,34,35,36,37]. In NSCLC, elevated MCV has been associated with poor survival in patients receiving radiotherapy for locally advanced disease, and as a prognostic marker, it has outperformed other erythrocyte indices [38]. However, the prognostic relevance and biological implications of MCV in patients undergoing curative-intent surgical resection remain unclear.

In this context, we investigated the independent association between preoperative MCV and overall survival (OS) in patients with surgically resected stage I–IIIA NSCLC. To elucidate the biological basis underlying the prognostic signal of MCV, we applied machine learning interpretability techniques, including least absolute shrinkage and selection operator regression (LASSO) and SHapley Additive exPlanations (SHAP), to dissect the relative contributions of individual hematologic parameters. Finally, given the established prognostic significance of systemic inflammatory markers, we investigated whether integrating MCV into the NUn score could enhance prognostic performance, thereby developing a composite biomarker. The NUn–MCV index captures both inflammatory burden and erythrocyte-derived physiological stress to enable refined risk stratification in resected NSCLC.

## 2. Materials and Methods

### 2.1. Study Population

A retrospective review was conducted on adult patients (≥18 years) with pathologically verified NSCLC who were treated with curative-intent resection at Kyung Hee University Hospital in Gangdong during the 2010–2023 study period. Preoperative staging was performed according to institutional standards and included contrast-enhanced chest and abdominopelvic computed tomography (CT) for anatomical assessment of the primary tumor and distant disease, as well as integrated positron emission tomography–CT (PET/CT) to evaluate metabolic activity of the primary lesion, regional lymph nodes, and potential extra-thoracic metastases.

Patients were eligible if they met the following criteria: (i) patients with stage I–II and select N2-negative stage IIIA NSCLC [39,40]; (ii) completion of standardized preoperative staging, including contrast-enhanced CT and PET/CT; (iii) curative-intent surgical resection with microscopically negative margins (R0) [41]; (iv) availability of complete baseline clinicopathologic and laboratory data obtained within 7 days before surgery; and (v) sufficient follow-up for survival analysis.

Patients were excluded if they had received neoadjuvant chemotherapy, radiotherapy, or immunotherapy before surgery; had stage IIIB or IV disease; had a concurrent malignancy or a history of another malignancy diagnosed within the preceding 5 years; or had an active infection or connective tissue disease requiring ongoing medical treatment at the time of surgery. These exclusions were applied to minimize confounding from treatment-related effects and non-cancer-related inflammatory conditions that could influence systemic biomarker levels.

Postoperatively, patients with stage II or IIIA disease were administered adjuvant chemotherapy. Surveillance was conducted using chest and abdominopelvic CT according to institutional standards.

The study protocol was approved by the Institutional Review Board (IRB) of Kyung Hee University Hospital in Gangdong (No. 2024-09-008), which waived the need for informed consent owing to the retrospective nature of the study.

### 2.2. Clinical Characteristics

The clinicopathological variables collected for analysis included demographic factors (age and sex), lifestyle factors (smoking history and alcohol consumption), and anthropometric and physiological parameters, including basal metabolic rate (BMR) and body mass index. Preoperative functional status was assessed using the American Society of Anesthesiologists Physical Status (ASA-PS) classification system. Treatment-related variables included the extent and type of surgical resection and the use of postoperative adjuvant therapy. Tumor-related variables included the histological subtype, primary tumor size, pathological stage, pleural invasion (PL), lymphatic invasion, vascular invasion, perineural invasion, and residual disease status. Alcohol consumption was defined as regular alcohol intake occurring more than once per week, regardless of quantity [42]. BMR was calculated using the Mifflin–St Jeor equation [43]. PL was categorized from 0 to 3 [44].

Laboratory assessments were performed as part of the routine preoperative evaluation and included hematologic indices and standard blood chemistry parameters. Hematologic measures included total white blood cell (WBC) count with differential counts, including absolute neutrophil, monocyte, and lymphocyte counts. Erythrocyte parameters included RBC count, hemoglobin concentration, hematocrit, and red-cell indices—MCV, mean corpuscular hemoglobin (MCH), and mean corpuscular hemoglobin concentration (MCHC). Platelet-related measures included platelet count and mean platelet volume.

Blood chemistry analyses encompassed markers of systemic inflammation, including CRP, as well as liver function and nutritional parameters, including total bilirubin, protein, albumin, aspartate aminotransferase, and alanine aminotransferase. The NUn score was calculated as follows: NUn score = 11.3894 + (0.005 × CRP [mg/L]) + (0.186 × WBC count [10^9^/L]) − (0.174 × albumin [g/L]) [45].

All laboratory measurements were obtained within 7 days before surgery, and when multiple values were available, the result closest to the operative date was used. Blood samples were processed within 1 h of venipuncture according to standardized institutional protocols. CBCs were collected in EDTA-anticoagulated tubes and analyzed using an automated impedance hematology analyzer (Beckman Coulter LH 1502; Beckman Coulter, Miami, FL, USA) [46,47].

### 2.3. Statistical Analysis

OS, the primary study endpoint, was calculated from the date of curative surgical resection to death from any cause, with censoring applied at the final follow-up. To preserve information content and avoid arbitrary dichotomization, all continuous variables were analyzed in their native continuous form. Missing data were addressed by complete-case analysis, with exclusion of patients who had missing values in covariates included in multivariable models. Given the low proportion of missing data, no imputation procedures were applied.

Associations between candidate variables and OS were examined using the Cox proportional hazards regression analysis. Variables demonstrating statistical significance in univariate analyses (*p* < 0.05) were entered into multivariate Cox models. Variance inflation factors (VIFs) were used to evaluate collinearity among predictors, whereas compliance with the proportional hazards assumption was formally assessed for each covariate, with non-conforming variables excluded from adjusted analyses. Log-relative hazard plots were generated to characterize continuous risk relationships beyond single hazard ratio estimates because conventional point estimates may inadequately reflect nonlinear risk gradients across the observed range. Potential interaction effects were evaluated using likelihood ratio tests.

To prioritize influential clinical and laboratory determinants, we applied LASSO regression, which performs coefficient shrinkage and data-driven variable selection within a unified modeling framework. This penalized regression framework enables simultaneous variable selection and coefficient shrinkage, thereby reducing overfitting and improving model parsimony. Model performance was quantified using the coefficient of determination and root mean squared error (RMSE), with the optimal regularization parameter identified via 10-fold cross-validation. Variables retaining nonzero coefficients in the optimized model were considered informative contributors and were subsequently corroborated using multivariate regression analyses.

To explore potential nonlinear associations and higher-order interactions not captured by linear modeling, an extreme gradient boosting (XGBoost) model was constructed. Model interpretability was achieved using SHAP, which quantifies each variable’s marginal contribution to model prediction while accounting for nonlinear and interaction effects. Variables with mean absolute SHAP values ≤0.01 were considered to exert negligible influence.

Discriminative performance was quantified using the concordance index (C-index) and integrated area under the curve (iAUC), with pairwise model comparisons conducted via 1000 bootstrap resamples. The incremental prognostic value was quantified using continuous net reclassification improvement (cNRI) and integrated discrimination improvement (IDI) at 3- and 5-year time points. Decision curve analysis (DCA) was conducted to evaluate the net clinical benefit across a range of threshold probabilities.

A prognostic nomogram was constructed based on the finalized multivariable model, and internal validation was performed using 1000 bootstrap resamples to evaluate robustness and predictive accuracy. Calibration was evaluated by comparing predicted and observed survival probabilities using calibration curves.

To benchmark the prognostic performance of the NUn–MCV index, separate multivariable Cox models were constructed by individually incorporating each composite biomarker—including the NUn–MCV index, NLR, LMR, MLR, CLR, CALLY index, HALP score, SII, SIRI, and inflammatory burden index (IBI)—into a common prognostic framework [10,14,16,17,48,49].

All statistical analyses were conducted using R software (version 4.4.0; R Foundation for Statistical Computing, Vienna, Austria). Statistical significance was defined as a two-sided *p* value < 0.05.

## 3. Results

### 3.1. Clinicopathological Characteristics of the Patients

A total of 427 patients with pathological stage I–IIIA NSCLC who underwent complete surgical resection were included in the final analysis. The baseline demographic, clinical, laboratory, and pathological characteristics of the study cohort are presented in Table 1. The median age at surgery was 68 years, and 59% of the patients were men. Lobectomy was the predominant surgical approach, and adenocarcinoma was the most frequently observed histological subtype. According to pathological staging, 302 patients (70.7%) had stage I disease, 66 (15.5%) had stage II disease, and 59 (13.8%) had stage IIIA disease. Preoperative laboratory assessments demonstrated substantial inter-individual variability across inflammatory and hematologic parameters. The median preoperative MCV was 91.6 fL (interquartile range [IQR], 89.0–94.5 fL) (Table 1).

### 3.2. Cox Regression Analysis for Predictors of OS

Over a median follow-up of nearly 5 years (58.0 months; IQR, 32.3–87.9 months), seven variables retained independent prognostic significance for OS in the adjusted Cox model: age, BMR, ASA-PS, stage, PL, MCV, and the NUn score. This set of predictors constituted Model 1 and achieved robust model fit and discrimination (C-index = 0.843; Akaike Information Criterion [AIC] = 792.71) (Table 2).

Next, we evaluated whether integrating MCV with the NUn score—comprising WBC count, albumin level, and CRP level—might enhance prognostic discrimination. A composite marker, termed the NUn–MCV index, was developed by linearly combining both variables according to their partial regression coefficients derived from the multivariate Cox model as follows: NUn–MCV index = (0.556 × NUn) + (0.063 × MCV).

Accordingly, the NUn–MCV index was incorporated in a second-round multivariate Cox regression analysis. In this model, six variables remained independent predictors of OS—age, BMR, ASA-PS, stage, PL, and the NUn–MCV index—and comprised Model 2 (C-index = 0.843, AIC = 790.71). All covariates satisfied the proportional hazards assumption and demonstrated minimal multicollinearity (VIF = 1.05–1.16), confirming model stability. The slightly lower AIC of Model 2 indicated a modest improvement in model fit compared with that of Model 1, while the overall discriminative performance (C-index) remained comparable. Based on these findings, Model 2 was finalized as the optimized prognostic model (Table 2).

Adjuvant chemotherapy was included in univariate analyses and demonstrated a significant association with overall survival. However, these variables did not remain independently significant in multivariable Cox regression and were therefore excluded from the final model.

Formal interaction testing revealed no significant effect modification by pathological stage for either MCV or the NUn–MCV index (*p* for interaction = 0.96 and 0.83, respectively), indicating that their associations with OS were consistent across disease stages.

### 3.3. Association Between Key Variables and Log-Relative Hazard in Predicting Survival

**Figure 1:**
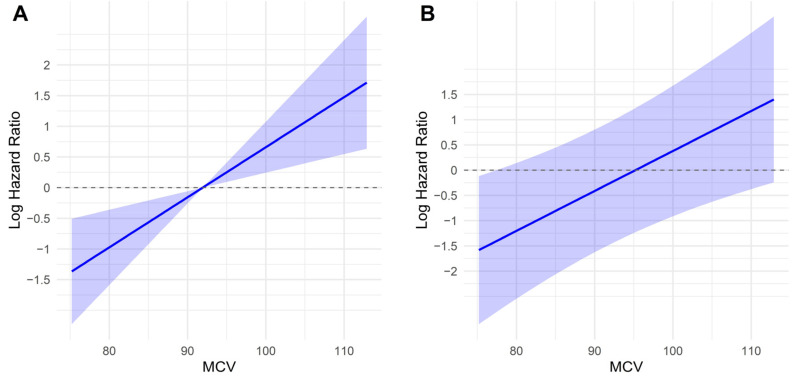

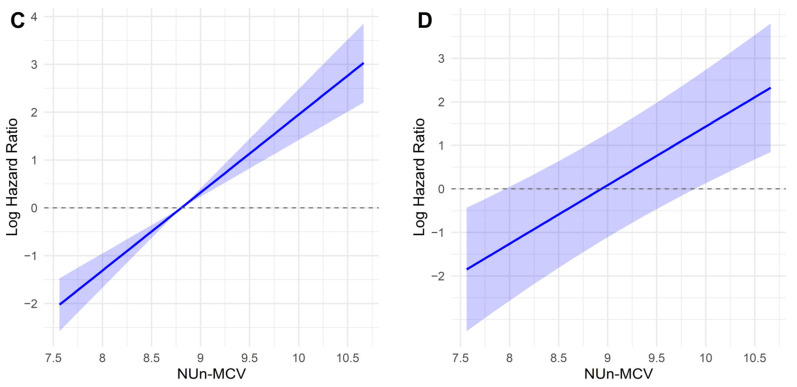
Association between key variables and log-relative hazards in predicting survival. Shaded areas represent 95% confidence intervals. (**A**) MCV, univariate analysis; (**B**) MCV, multivariate analysis; (**C**) NUn–MCV, univariate analysis; (**D**) NUn–MCV, multivariate analysis.

Unadjusted analyses revealed a dose–response relationship between MCV and mortality risk, characterized by a linear increase in the log-relative hazard with increasing MCV (Figure 1A). Adjustment for established clinical and pathological factors did not materially alter this pattern, indicating that MCV conveys prognostic information that is independent of conventional risk determinants and functions as a continuous survival marker in surgically treated NSCLC (Figure 1B).

Consistent findings were observed for the NUn–MCV index. In the univariate analysis, higher index values were linearly associated with increased log-relative hazard, reflecting inferior survival outcomes (Figure 1C). This dose–response relationship persisted after multivariable adjustment for age, BMR, ASA-PS, stage, and PL, thereby confirming the NUn–MCV index as a continuous, independent prognostic determinant of OS (Figure 1D).

### 3.4. Variables Contributing to Key Variables Such as MCV and the NUn–MCV Index

To delineate the biological and statistical determinants of MCV and the NUn–MCV index, we applied complementary linear (LASSO, multiple linear regression) and nonlinear (XGBoost with SHAP) modeling approaches.

The variation in MCV was explained almost exclusively by intrinsic erythrocyte parameters—MCH and MCHC. LASSO regression demonstrated a near-perfect model fit (R^2^ = 0.9933; RMSE = 0.4224), whereas XGBoost showed a substantially inferior performance (RMSE = 2.1893), indicating that MCV is governed predominantly by linear relationships. Consistently, both LASSO and multiple linear regression identified MCH and MCHC as the only variables with large, independent, and highly significant effects (both *p* < 0.001).

**Figure 2:**
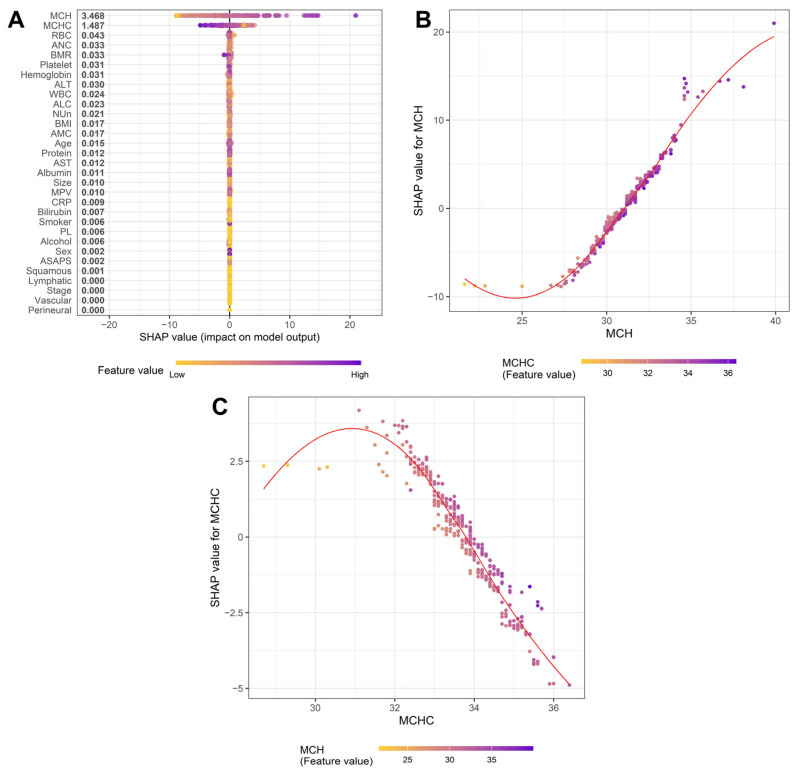
SHAP summary for XGBoost models predicting MCV. (**A**) SHAP summary plot, (**B**) MCH dependence plot, and (**C**) MCHC dependence plot.

SHAP analysis confirmed MCH and MCHC as the dominant contributors to MCV, with all other variables exerting a negligible influence (Figure 2A). A high MCH exerted a strong positive effect on MCV, particularly in the setting of elevated MCHC, whereas a high MCHC produced a sharp negative contribution, which was most pronounced when MCH was also high (Figure 2B–C). Collectively, these findings indicate that the MCV reflects the net balance of two opposing erythroid processes: cellular enlargement driven by increased hemoglobin content (MCH) and membrane-level constraints reflected by MCHC.

The NUn–MCV index exhibited a structurally self-contained architecture driven almost entirely by the additive effects of the NUn score and MCV. Only these two components retained nonzero coefficients in LASSO models, and XGBoost showed inferior performance relative to linear models (RMSE = 0.0944 vs. 0.0155), indicating a minimal contribution from higher-order interactions. Multiple linear regression analysis confirmed their independent prognostic contributions (both *p* < 0.001), and likelihood ratio testing demonstrated no meaningful interaction between them.

**Figure 3:**
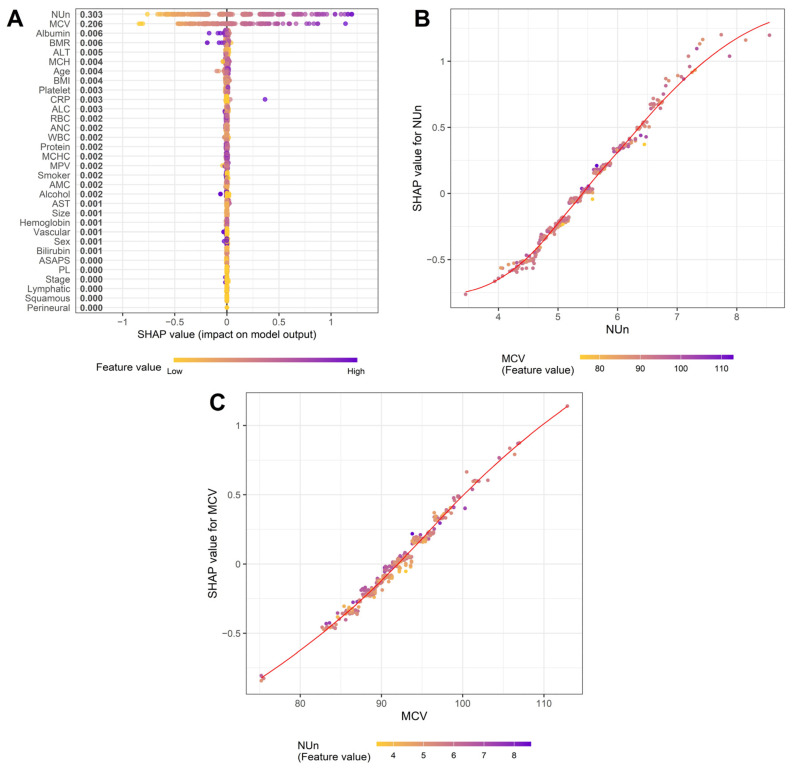
SHAP summary for XGBoost models predicting the NUn–MCV index. (**A**) SHAP summary plot, (**B**) NUn score dependence plot, and (**C**) MCV dependence plot.

SHAP analysis reinforced these findings by identifying the NUn score and MCV as exclusive drivers of the composite index (Figure 3A). The NUn score showed a monotonic sigmoidal relationship with survival prediction, with adverse effects at low values and a sharply increasing risk at higher values, particularly when accompanied by elevated MCV (Figure 3B). In contrast, the MCV exhibited an approximately linear relationship across the observed range, with only a modest interaction with the NUn score (Figure 3C). Together, these results indicate that the NUn–MCV index integrates systemic inflammatory burden and intrinsic erythroid stress in an additive, biologically interpretable manner, with nonlinear effects confined to extreme physiological states.

### 3.5. Comparison Between the Full, Baseline, and Intermediate Models

The full model (FM), which incorporated the NUn–MCV index in addition to the clinical covariates specified in Model 2, demonstrated superior discriminative performance compared to both the baseline model (BM), which was based solely on pathological stage, and the intermediate model (IM), which included the same clinical covariates as the FM but did not incorporate the NUn–MCV index. The FM achieved a significantly higher C-index than the BM (0.843 vs. 0.691; *p* < 0.001) and a numerically higher value than the IM (0.843 vs. 0.826; *p* = 0.058). Consistent results were observed for the iAUC, with the FM outperforming both the BM (0.812 vs. 0.663; *p* < 0.001) and IM (0.812 vs. 0.799; *p* < 0.001) (Table 3).

The cNRI analysis demonstrated significantly superior risk reclassification with the FM compared to that with the BM at 3 years (cNRI = 0.514, *p* < 0.001) and 5 years (cNRI = 0.418, *p* < 0.001). The FM also outperformed the IM, yielding significant reclassification gains at 3 years (cNRI = 0.301, *p* = 0.010) and 5 years (cNRI = 0.187, *p* = 0.044). Consistent with these findings, IDI analyses favored the FM over the BM at both 3 years (IDI = 0.265, *p* < 0.001) and 5 years (IDI = 0.245, *p* < 0.001). Relative to the IM, the FM also demonstrated significant improvements in discrimination at 3 years (IDI = 0.073, *p* = 0.004) and 5 years (IDI = 0.050, *p* = 0.022). Collectively, these results indicate that incorporation of the NUn–MCV index confers significant incremental improvements in both risk reclassification and discrimination beyond conventional clinical predictors (Table 3).

**Figure 4:**
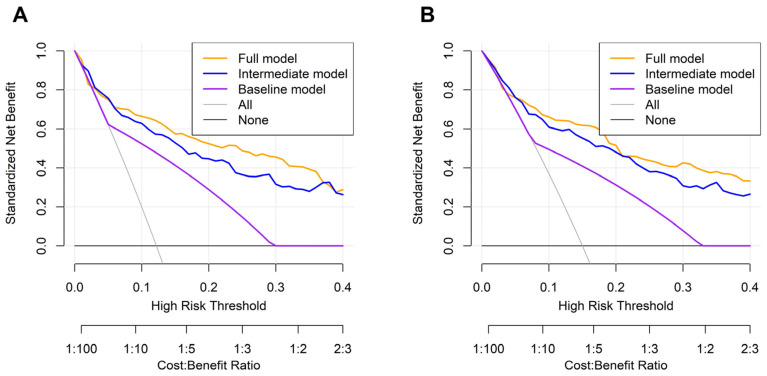
Decision curve analysis comparing the full model with baseline and intermediate models for predicting 3- (**A**) and 5-year (**B**) overall survival.

DCAs at 3 years showed that incorporation of the NUn–MCV index conferred a meaningful net clinical benefit over conventional models across most threshold probabilities, with limited incremental value only at extreme risk thresholds where prognosis was largely driven by established clinical factors (Figure 4A). At 5 years, the FM maintained a superior net benefit across most clinically relevant thresholds; however, convergence with the IM was observed around a threshold probability of approximately 0.20, indicating threshold-dependent attenuation of the incremental prognostic contribution of the NUn–MCV index (Figure 4B).

### 3.6. Nomogram for Predicting 3- and 5-Year Survival Using the FM

**Figure 5:**
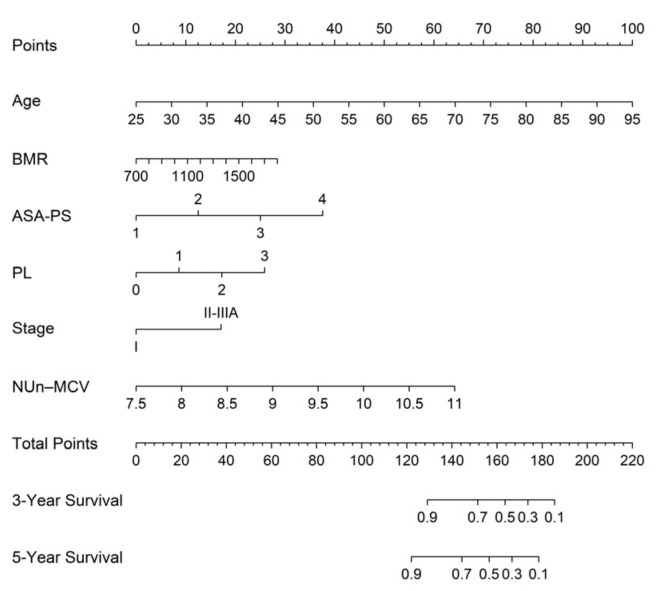
Predictive nomogram for overall survival based on the full model.

A nomogram based on the FM, incorporating age, BMR, ASA-PS, stage, PL, and the NUn–MCV index, was constructed to estimate individualized probabilities of 3- and 5-year OS. For each variable, points were assigned according to their relative contribution to the prognosis, and the total points corresponded to the predicted survival probabilities at 3 and 5 years (Figure 5).

**Figure 6:**
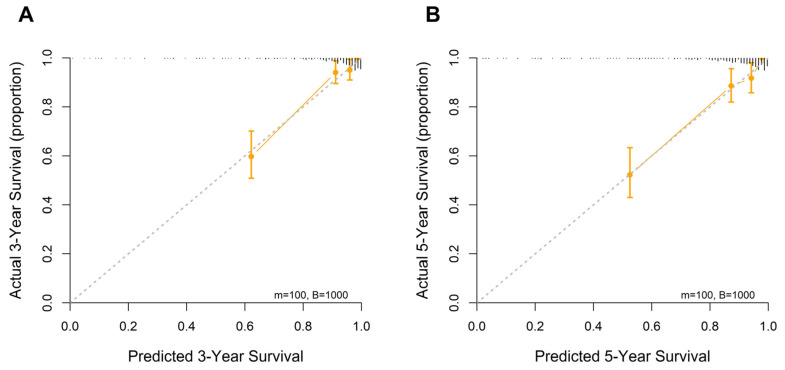
Calibration curve analysis for predicting 3- (**A**) and 5-year (**B**) overall survival based on the full model.

Calibration curves demonstrated close concordance between the predicted and observed survival probabilities at all evaluated time points, indicating accurate risk estimation and satisfactory calibration of the nomogram. Model calibration was assessed using bootstrap-corrected calibration plots generated using the rms package, with approximately 100 patients per calibration group (m = 100) and 1000 bootstrap resamples (B = 1000) (Figure 6).

### 3.7. The NUn–MCV Index vs. Established Biomarkers

To benchmark the prognostic discrimination of the NUn–MCV index against established inflammation- and nutrition-based composite biomarkers, separate multivariable Cox proportional hazards models were fitted by individually incorporating each biomarker (NLR, LMR, MLR, CLR, CALLY index, HALP score, SII, SIRI, and IBI) into a common prognostic platform (the IM) that included age, BMR, ASA-PS, stage, and PL.

Among all evaluated models, incorporation of the NUn–MCV index achieved the highest discriminative performance, yielding a C-index of 0.843. The model augmented with the CALLY index also demonstrated a strong prognostic discrimination (C-index = 0.837). In contrast, models incorporating the IBI, CLR, MLR, SIRI, NLR, HALP score, LMR, or SII produced comparatively smaller gains in discrimination, with C-indices ranging from 0.827 to 0.831. Importantly, the NUn–MCV-augmented model outperformed models incorporating either component alone, including the NUn score (C-index = 0.841) and MCV (C-index = 0.829), supporting the additive prognostic value of the composite index.

**Figure 7:**
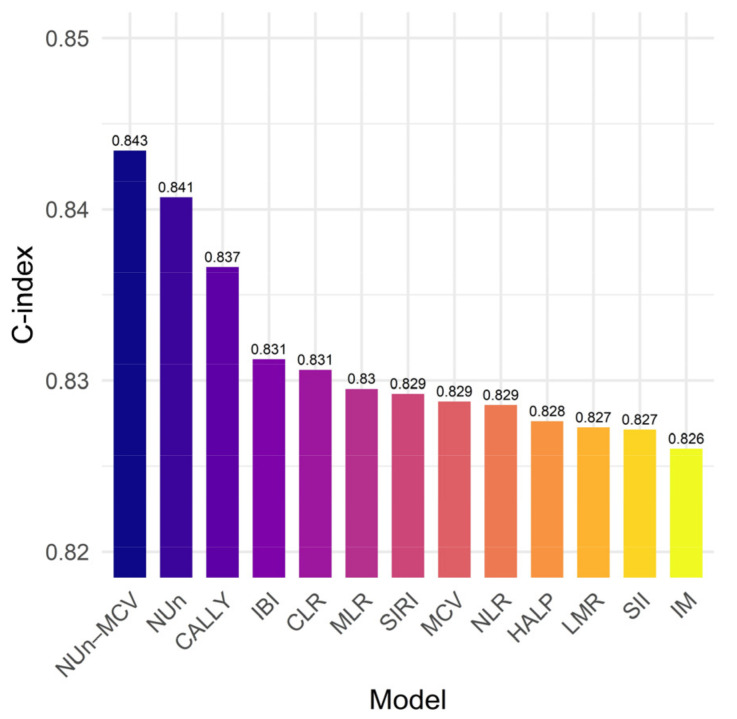
NUn–MCV vs. established biomarkers.

Collectively, these results indicate that although multiple inflammation- and nutrition-based biomarkers enhance prognostic stratification in resected NSCLC, the NUn–MCV index provides the most robust and consistent improvement in model discrimination across all evaluated biomarkers (Figure 7).

## 4. Discussion

In this study, we demonstrated that preoperative MCV is an independent prognostic determinant of OS in patients with stage I–IIIA NSCLC treated with curative-intent resection. Furthermore, combining MCV with the NUn score to form the composite NUn–MCV index yielded an incremental prognostic value beyond conventional clinicopathological variables. An FM incorporating the NUn–MCV index along with age, BMR, ASA-PS, stage, and PL achieved superior discrimination compared with both the BM and IM. These findings indicate that erythrocyte-derived parameters provide clinically meaningful prognostic information and support the NUn–MCV index as a practical biomarker for the refined risk stratification of resected NSCLC.

Elevated MCV has been linked to adverse outcomes across a range of malignancies, including cancers of the head and neck, esophagus, stomach, and colorectum [32,33,34,35,36,37]. In NSCLC, previous studies have shown that a higher MCV is associated with poorer survival in patients treated with radiotherapy for locally advanced disease [38]. Extending these observations, our results establish that the MCV has prognostic relevance in curative surgical resection and across early to locally advanced stages. Importantly, the observed linear association between MCV and mortality risk persisted after adjusting for established clinical and pathological covariates.

Mechanistic analyses provided insight into the biological underpinnings of this association. Linear modeling using LASSO regression revealed that MCV variability was almost entirely explained by intrinsic erythrocyte indices—specifically MCH and MCHC—but showed no meaningful association with CRP level, albumin level, WBC count, or major clinical variables. SHAP analysis further confirmed the dominant and opposing contributions of MCH and MCHC to MCV. These findings reinforce the biological plausibility of MCV as an integrated reflection of erythroid physiology rather than as a nonspecific surrogate of systemic inflammation or comorbidity. Consistent with this interpretation, nonlinear modeling using XGBoost offered no performance advantage over linear approaches (LASSO regression), indicating that higher-order interactions contributed minimally to the prognostic signal captured by MCV.

From a biological perspective, elevated MCV may reflect maladaptive systemic responses to tumor-associated hypoxia. Rapid tumor growth in NSCLC frequently generates a hypoxic microenvironment, leading to stabilization of hypoxia-inducible factor–1α and subsequent upregulation of erythropoietin (EPO) production [50,51]. Sustained EPO stimulation can accelerate erythropoiesis and promote the premature release of large immature erythrocytes into the circulation, resulting in increased MCV [52,53]. Hypoxia-driven signaling pathways also facilitate aggressive tumor behavior, including epithelial–mesenchymal transition, enhanced invasiveness, and metastatic potential [50,53]. In this context, elevated MCV may serve as a systemic marker of an underlying tumor microenvironment that is already permissive for progression and recurrence [50,54].

In parallel, cancer-associated oxidative stress can impair erythrocyte membrane integrity and deformability [55]. Enlarged and less deformable erythrocytes may compromise microvascular flow, further limiting oxygen delivery within the tumor microcirculation and exacerbating hypoxia [50,53,55]. This vicious cycle may contribute not only to tumor progression but also to resistance to adjuvant therapies, as hypoxic tumor regions are known to exhibit reduced sensitivity to both radiotherapy and chemotherapy [50,52]. Consequently, even after complete resection, patients with elevated MCV may harbor residual tumor cells within a biologically unfavorable microenvironment, leading to inferior long-term outcomes.

Although hypoxia-related erythroid stress represents a biologically plausible mechanism linking elevated MCV to adverse outcomes in NSCLC, the present study cannot fully exclude nutritional deficiencies as contributory factors. Vitamin B12 and folate deficiencies are well-established causes of macrocytosis and were not directly assessed in this retrospective cohort. Accordingly, residual confounding related to unmeasured nutritional status remains possible. Nevertheless, the association between preoperative MCV and OS persisted after comprehensive adjustment for demographic, clinical, pathological, and inflammation-based variables, and mechanistic analyses indicated that MCV variability was primarily driven by intrinsic erythrocyte indices rather than systemic inflammatory markers. These findings suggest that MCV captures biologically relevant alterations in erythroid physiology; however, prospective studies incorporating detailed nutritional and metabolic profiling are warranted to further delineate the relative contributions of hypoxia-related mechanisms and nutritional determinants.

Although MCV independently predicted survival, its effect size was modest. To enhance prognostic discrimination, we constructed the NUn–MCV index by linearly combining MCV with the NUn score [45]. The absence of a significant interaction between MCV and the NUn score suggests additive rather than synergistic effects, reflecting the integration of distinct biological domains: erythroid stress and systemic inflammation.

Supporting this interpretation, LASSO regression demonstrated that the NUn–MCV index is structurally self-contained, with its two constituent components retaining nonzero coefficients among all candidate variables. Linear models consistently outperformed nonlinear approaches, indicating that the index is governed by a simple additive structure with minimal susceptibility to confounding interactions. These characteristics enhance the robustness, interpretability, and clinical applicability of the NUn–MCV index as a prognostic tool.

Biologically, the NUn–MCV index integrates complementary dimensions of host vulnerability. The NUn score captures systemic inflammatory burden, immune activation, and nutritional status through CRP level, albumin level, and WBC count, whereas the MCV reflects erythropoietic efficiency, red-cell metabolic state, and physiological stress. Together, the composite index provides a coherent representation of host inflammatory and hematologic resilience, which may be particularly relevant in perioperative and postoperative settings.

In addition to the NUn–MCV index, several established clinical and pathological variables, including age, BMR, ASA-PS, stage, and PL, remained independently associated with OS. Advanced age reflects diminished physiological reserve and increased comorbidity burden, which adversely influence long-term outcomes [11,56,57,58].

BMR, representing the basal energy expenditure required for essential physiological functions, has recently emerged as a prognostic factor in oncology [59,60]. Our findings extend this concept to NSCLC and identify BMR as an independent predictor of survival after curative resection. To the best of our knowledge, this is the first study to report such an association in NSCLC, although warranting further validation. Notably, BMR remained an independent prognostic factor in multivariable analysis, whereas BMI did not. Although BMR is calculated using age, sex, height, and weight, its prognostic relevance in this context is unlikely to reflect anthropometric characteristics alone, as age and sex were already adjusted for in the model. Instead, BMR may capture disease-related hypermetabolism or catabolic stress associated with malignancy, reflecting increased energy expenditure and metabolic burden that are not adequately represented by BMI. In contrast, BMI is a static measure of body composition and may fail to account for dynamic metabolic alterations accompanying cancer progression. These findings suggest that metabolic dysregulation, rather than body size per se, contributes meaningfully to survival heterogeneity in resected NSCLC. In addition, the persistence of BMR as a prognostic variable underscores the clinical relevance of metabolic demand and energy imbalance as components of host vulnerability in oncologic outcomes.

ASA-PS provided additional prognostic information, consistent with its role as a global indicator of preoperative physiological vulnerability and its established association with adverse outcomes in NSCLC [56]. Pathological stage remained the dominant determinant of survival, underscoring the central role of tumor burden and disease extent, whereas PL reflected biologically aggressive behavior and an increased risk of recurrence [11,14,48,57,58]. Importantly, multicollinearity was minimal and all covariates satisfied the proportional hazards assumption, supporting the internal validity of the multivariable model. The lack of independent significance of adjuvant therapy in multivariable analysis likely reflects its strong correlation with pathological stage and other postoperative risk factors rather than the absence of therapeutic benefit.

Integrating these variables into the FM yielded meaningful improvements in discrimination and risk stratification compared with integrating them into the BM or IM. Although the absolute improvements in discrimination and reclassification metrics achieved by incorporating the NUn–MCV index were numerically modest, such gains are consistent with those reported for many clinically adopted prognostic biomarkers in oncology. Importantly, these improvements may be clinically meaningful in the context of postoperative NSCLC management, where therapeutic and surveillance decisions are frequently made within broad pathological stage categories. By refining risk stratification among stage-matched patients, the NUn–MCV index may help identify individuals with disproportionately high biological risk who could benefit from closer surveillance, earlier detection of recurrence, or more informed discussions regarding adjuvant treatment in borderline clinical scenarios. Given that the NUn–MCV index is derived entirely from routinely available laboratory parameters, its integration into clinical workflows may enhance individualized postoperative management without additional cost or procedural burden.

When benchmarked against established inflammation- and nutrition-based biomarkers, including the CALLY index, IBI, CLR, MLR, SIRI, NLR, HALP score, LMR, and SII, the NUn–MCV-augmented model demonstrated the highest discriminative performance. Notably, the composite index outperformed models incorporating either MCV or the NUn score alone, reinforcing the additive prognostic value of integrating erythrocyte-derived and inflammatory information.

This study offers several conceptual, methodological, and clinical strengths. First, it identifies preoperative MCV—a universally available and inexpensive hematologic parameter—as an independent prognostic marker in patients with stage I–IIIA NSCLC undergoing curative-intent resection. By demonstrating a linear and continuous association with OS, this study avoids arbitrary dichotomization and preserves prognostic information, thereby enhancing interpretability and generalizability. Second, the integration of MCV with the NUn score to derive the composite NUn–MCV index represents a biologically coherent and statistically justified advancement. Rather than relying solely on inflammation-based metrics, the composite index integrates complementary domains of host vulnerability—systemic inflammation and erythroid physiology—resulting in improved discriminative performance across multiple validation metrics. Importantly, model selection was guided by objective criteria (AIC), strengthening the methodological rigor of the model-building process. Third, this study moves beyond conventional association analysis by incorporating machine-learning–based interpretability (LASSO and SHAP), enabling mechanistic decomposition of MCV into its principal erythrocyte determinants (MCH and MCHC). This biologically granular insight distinguishes the work from prior prognostic studies and reframes macrocytosis in NSCLC as a marker of altered erythroid homeostasis rather than a nonspecific surrogate of comorbidity. Fourth, robustness was reinforced through comprehensive internal validation using 1000 bootstrap resamples, formal assessment of model assumptions, evaluation of multicollinearity, and analysis of continuous variables without categorization. These features enhance statistical reliability and reduce overfitting. Finally, the proposed biomarker and composite index are immediately translatable to clinical practice, as they rely exclusively on routinely obtained laboratory parameters without additional cost or testing burden. This practical applicability increases the likelihood of real-world implementation and facilitates refined postoperative risk stratification within anatomically defined stage groups.

This study had several limitations that merit consideration. First, this was a single-center, retrospective analysis conducted in a predominantly Asian population, which may restrict the generalizability of the findings to other ethnic or geographic groups. Baseline hematologic indices, inflammatory profiles, and perioperative management strategies may vary across populations and healthcare systems, potentially influencing effect estimates or optimal risk thresholds. Nevertheless, MCV represents a fundamental erythrocyte parameter with well-established physiological underpinnings, suggesting that its association with survival may be biologically conserved across populations. Accordingly, external validation in independent, multi-center cohorts with diverse ethnic backgrounds is essential to confirm the robustness, transportability, and clinical applicability of the NUn–MCV index.

Second, despite adjustment for multiple clinical and laboratory parameters, certain conditions known to influence MCV—particularly vitamin B12 or folate deficiency and thyroid dysfunction—were not directly captured in this retrospective cohort and may represent sources of residual confounding. Nevertheless, the prognostic association between preoperative MCV and OS remained robust after comprehensive adjustment for demographic, clinical, pathological, and inflammation-based variables, suggesting that MCV conveys biologically relevant information not fully accounted for by these factors. Moreover, mechanistic analyses indicated that variability in MCV was predominantly driven by intrinsic erythrocyte indices rather than systemic inflammatory markers, supporting the interpretation of MCV as a marker of altered erythroid physiology rather than a nonspecific surrogate of comorbidity. Finally, although the contribution of unmeasured nutritional or metabolic determinants cannot be entirely excluded, prospective studies incorporating detailed assessments of vitamin status and thyroid function are warranted to further elucidate the biological basis of MCV-associated risk.

Third, the relatively high proportion of stage I disease may have contributed to favorable survival outcomes; however, the pathological stage was rigorously adjusted for and remained an independent predictor in multivariable analyses. Fourth, the proposed biological mechanisms, particularly those related to iron metabolism and hypoxia-driven erythropoiesis, were inferred indirectly from red-cell indices. Future studies incorporating direct measurements of vitamin B_12_, folate, hepcidin, EPO, and hypoxia-related biomarkers are required to validate these mechanistic hypotheses. Fifth, the analysis was limited to baseline preoperative laboratory values, which precluded assessment of longitudinal changes in the NUn–MCV index over the course of the disease or in response to treatment. Sixth, despite extensive internal validation, the absence of external validation is an important limitation that should be addressed in future prospective studies. Finally, competing risks such as non-cancer-related mortality were not formally evaluated. Because cause-specific death could not be consistently determined in this retrospective cohort, OS—ascertained through complete national registry data—was selected as the primary endpoint.

## 5. Conclusions

In patients with stage I–IIIA NSCLC treated with curative-intent surgery, preoperative MCV independently stratified OS risk after adjusting for established clinicopathological variables. The incorporation of MCV into the NUn score to form the NUn–MCV index provided meaningful incremental prognostic information beyond the established clinical and pathological factors. A multivariable prognostic model integrating the NUn–MCV index with conventional variables achieved superior discriminative performance compared with models based on stage alone (the BM) or standard clinical parameters (the IM).

The NUn–MCV index is a biologically interpretable and readily accessible biomarker that integrates erythrocyte-derived physiological stress with the systemic inflammatory status. Given its reliance on routinely available preoperative laboratory data, this composite index may facilitate refined risk stratification, inform perioperative decision-making, and support individualized postoperative management of resected NSCLC. A prospective evaluation of external independent cohorts is necessary to establish the generalizability and clinical relevance of this approach.

## Figures and Tables

**Table 1:** Clinicopathological characteristics of the patients.

Variables		*n* (%) or Median (IQR)	Variables		*n* (%) or Median (IQR)
Age, years		68 (12)	Vascular invasion		
Sex				Yes	24 (5.6%)
	Men	252 (59.0%)		No	403 (94.4%)
	Women	175 (41.0%)	Perineural invasion		
Smoking				Yes	7 (1.6%)
	Current/Past	185 (43.3%)		No	420 (98.4%)
	Never	242 (56.7%)	TNM stage		
Alcohol consumption				IA/IB	302 (70.7%)
	Yes	109 (25.5%)		IIA/IIB/IIIA	125 (29.3%)
	No	318 (74.5%)	Adjuvant therapy		
BMR, kcal/day		1256.0 (338.5)		Yes	105 (24.6%)
BMI, kg/m^2^		23.8 (4.2)		No	322 (75.4%)
ASA-PS			WBC, ×10^3^ per μL		6.3 (2.2)
	1/2	351 (82.2%)	ANC, ×10^3^ per μL		3.6 (1.7)
	3/4	76 (17.8%)	AMC, ×10^3^ per μL		0.5 (0.2)
Resection			ALC, ×10^3^ per μL		1.8 (0.7)
	Sublobar resection	136 (31.9%)	RBC, ×10^6^ per μL		4.3 (2.0)
	Lobectomy	280 (65.6%)	Hemoglobin, g/dL		13.2 (2.1)
	Bilobectomy	5 (1.2%)	Hematocrit, %		39.0 (5.9)
	Pneumonectomy	6 (1.4%)	MCV, fL		91.6 (5.5)
Histology			MCH, pg		30.9 (2.1)
	Squamous	98 (23.0%)	MCHC, g/dL		33.7 (1.3)
	Non-squamous	329 (77.0%)	Platelet, ×10^6^ per μL		0.2 (0.1)
Tumor size, cm		2.5 (1.8)	MPV, fL		9.6 (1.1)
Pleural invasion (PL)			Protein, g/dL		7.2 (0.7)
	0	329 (77.0%)	Albumin, g/dL		4.2 (0.5)
	≥1	98 (23.0%)	Total bilirubin, mg/dL		0.5 (0.3)
Lymphatic invasion			AST, U/L		22 (8)
	Yes	55 (12.9%)	ALT, U/L		16 (11)
	No	372 (87.1%)	C-reactive protein, mg/L		1 (2)

ASA-PS, American Society of Anesthesiologists physical status; BMI, body mass index; BMR, basal metabolic rate; IQR, interquartile range; MCV, mean corpuscular volume; MPV, mean platelet volume; TNM, tumor-node-metastasis.

**Table 2:** Multivariate Cox regression analysis for predictors of overall survival.

Variables	Model 1 HR (95% CI)	*p* Value	Model 2 HR (95% CI)	*p* Value
Age, years	1.08 (1.05–1.11)	<0.001	1.08 (1.05–1.11)	<0.001
BMR, kcal/day	1.00 (1.00–1.00)	0.022	1.00 (1.00–1.00)	0.022
ASA-PS †	1.98 (1.32–2.99)	0.001	1.98 (1.32–2.99)	0.001
Pleural invasion (PL) †	1.60 (1.28–2.00)	<0.001	1.60 (1.28–2.00)	<0.001
TNM stage (II/IIIA vs. I)	2.55 (1.60–4.06)	<0.001	2.55 (1.60–4.05)	<0.001
MCV, fL	1.07 (1.02–1.12)	0.008	-	-
NUn score	1.74 (1.34–2.27)	<0.001	-	-
NUn–MCV index	-	-	2.72 (1.74–4.25)	<0.001

† ordinal variable. Hazard ratios for continuous variables are reported per one-unit increase (e.g., per 1-year increase in age, per 1 kcal/day increase in BMR, per 1 fL increase in MCV, and per 1-unit increase in NUn score). The C-indices (95% CI) for Models 1 and 2 were identical at 0.843 (0.801–0.886), indicating a comparable discriminative performance. Model 2 had a slightly lower AIC (790.71 vs. 792.71), suggesting a modest improvement in the model fit. NUn, Noble and Underwood.

**Table 3:** Comparison of the full model with the baseline and intermediate models for predicting survival outcomes.

Metrics	Baseline Model (BM)	Intermediate Model (IM)	Full Model (FM)	Gain (FM vs. BM)	*p* Value	Gain (FM vs. IM)	*p* Value
C-index	0.691 (0.028)	0.826 (0.023)	0.843 (0.022)	0.155 (0.023)	<0.001	0.018 (0.011)	0.058
iAUC	0.663 (0.027)	0.799 (0.023)	0.812 (0.023)	0.149 (0.009)	<0.001	0.015 (0.004)	<0.001
cNRI 3 Y				0.514 (0.078)	<0.001	0.301 (0.091)	0.010
cNRI 5 Y				0.418 (0.075)	<0.001	0.187 (0.087)	0.044
IDI 3 Y				0.265 (0.046)	<0.001	0.073 (0.033)	0.004
IDI 5 Y				0.245 (0.044)	<0.001	0.050 (0.031)	0.022

The values in parentheses are standard errors. The FM consists of age, BMR, ASA-PS, stage, PL, and NUn–MCV. The BM relies solely on the stage. The IM consists of the same variables as the FM, except for NUn–MCV. Y, year.

## Data Availability

The data supporting the findings of this study are available from the corresponding author upon reasonable request and are subject to ethical and institutional restrictions.

## Abbreviations

The following abbreviations are used in this manuscript:

ASA-PS	American Society of Anesthesiologists Physical Status
CALLY	CRP–albumin–lymphocyte
HALP	Hemoglobin–albumin–lymphocyte–platelet
IBI	Inflammatory burden index
LASSO	Least absolute shrinkage and selection operator
PL	Pleural invasion
SHAP	SHapley Additive exPlanations
SII	Systemic immune-inflammation index
SIRI	Systemic inflammation response index
XGBoost	Extreme gradient boosting
